# Unveiling *Trichosporon austroamericanum* sp. nov.: A Novel Emerging Opportunistic Basidiomycetous Yeast Species

**DOI:** 10.1007/s11046-024-00851-4

**Published:** 2024-05-06

**Authors:** Elaine C. Francisco, Marie Desnos-Ollivier, Chendo Dieleman, Teun Boekhout, Daniel Wagner de C. L. Santos, José O. Medina-Pestana, Arnaldo L. Colombo, Ferry Hagen

**Affiliations:** 1 Department of Medical Mycology, Westerdijk Fungal Biodiversity Institute (WI-KNAW), Uppsalalaan 8, 3584CT Utrecht, The Netherlands; 2 Division of Infectious Diseases, Escola Paulista de Medicina-Universidade Federal de São Paulo, São Paulo, Brazil; 3 Département de Mycologie, Centre National de Référence des Mycoses invasives et Antifongiques, Institut Pasteur, Université de Paris Cité, Paris, France; 4 College of Science, King Saud University, 11451 Riyadh, Saudi Arabia; 5 Instituto D´Or de Pesquisa e Ensino, IDOR, São Luís, Maranhão Brazil; 6 Serviço de Nefrologia, Hospital do Rim, Universidade Federal de São Paulo, São Paulo, Brazil; 7 Antimicrobial Resistance Institute of São Paulo (ARIES), São Paulo, Brazil; 8 Institute of Biodiversity and Ecosystem Dynamics (IBED), University of Amsterdam, Amsterdam, The Netherlands; 9 Department of Medical Microbiology, University Medical Center Utrecht, Utrecht, The Netherlands

**Keywords:** Basidiomycetous yeast, *Trichosporon*, Invasive trichosporonosis, Taxonomy, Emerging pathogen

## Abstract

During an epidemiological survey, a potential novel species within the basidiomycetous yeast genus *Trichosporon* was observed. The clinical strain was obtained from a urine sample taken from a Brazilian kidney transplant recipient. The strain was molecularly identified using the intergenic spacer (IGS1) ribosomal DNA locus and a subsequent phylogenetic analysis showed that multiple strains that were previously reported by other studies shared an identical IGS1-genotype most closely related to that of *Trichosporon inkin*. However, none of these studies provided an in-depth characterization of the involved strains to describe it as a new taxon. Here, we present the novel clinically relevant yeast for which we propose the name *Trichosporon austroamericanum* sp. nov. (holotype CBS H-24937). *T. austroamericanum* can be distinguished from other siblings in the genus *Trichosporon* using morphological, physiological, and phylogenetic characters.

**Supplementary Information:**

The online version contains supplementary material available at 10.1007/s11046-024-00851-4.

## Introduction

The genus *Trichosporon* encompasses anamorphic basidiomycetous yeast-like fungi recognized for their remarkable adaptations to different hosts and habitats, including plants, soils, mammals, and human beings [1–4]. Particularly in human hosts, these yeasts may be part of the commensal microbiota and some species can cause a wide range of superficial and invasive infections in susceptible patients [5–8].

Since its first description in 1865 by the German physician Hermann Beigel, the taxonomy of *Trichosporon* has undergone extensive revisions. Initially, the genus contained both ascomycetous and basidiomycetous yeasts that asexually reproduced with arthroconidia [4, 9]. For several decades, the systematics of this genus heavily relied on morphological, biochemical, and physiological characteristics, resulting in potential conflicts in genus and species identification [4, 10–13]. In 2015, a phylogenetic classification framework was proposed for the families and genera within the *Tremellomycetes*, resulting in substantial steps toward understanding the taxonomy of *Trichosporon*, especially in terms of differentiating at genus and species levels [14, 15]. Consequently, most of the 51 species previously classified as *Trichosporon* species were reclassified or accommodated into proposed or amended genera within the *Trichosporonaceae* family, such as *Cutaneotrichosporon*, *Apiotrichum*, *Haglerozyma*, *Effuseotrichosporon* and *Pascua* [14–17].

Currently, the *Trichosporon* genus comprises twelve species, with six of them capable of causing a diverse range of manifestations in human hosts, including superficial mycoses (e.g., white piedra, onychomycosis), summer-type hypersensitivity pneumonitis, esophageal lesions, fungemia, endocarditis, and other deep-seated infections [3–5, 7, 14, 16–18]. *Trichosporon asahii* is the predominant species causing human invasive trichosporonosis, followed by *Trichosporon inkin* and *Trichosporon asteroides* [2, 3, 6, 8, 19].

Globally, *T. inkin* has been reported as the second or third most common species causing invasive trichosporonosis in studies published by authors from Brazil, China, Greece, Spain, and India [3, 20–23]. Of note, *T. inkin* shares close phylogenetic characteristics with *T. asahii*, displaying a high capability for epithelial adherence and production of robust biofilm communities with enhanced tolerance to antifungal drugs [17, 24–26].

In this study we describe a novel yeast species closely related to *T. inkin*, for which we proposed the name *Trichosporon austroamericanum*, a fungal pathogen cultured from a urine sample obtained by an ultrasound-guided percutaneous puncture performed in a Brazilian kidney transplant recipient with an abdominal collection secondary to urinary fistula.

## Material and Methods

### Case Description

The yeast culture L9630 (= CBS 17435, ex-type) was initially isolated in 2013 from a urine sample collected intraoperatively during a surgery performed to correct a urinary fistula complicating the ureterovesical anastomosis of a 15-year-old female kidney transplant patient who was hospitalized at the Kidney and Hypertension Hospital in São Paulo, Brazil.

Fungal elements were abundantly observed in the urine sediment during a wet-mount examination, and the sample was subsequently cultured on routine microbiological media, including Sabouraud dextrose agar (Difco, BD, Franklin Lakes, NJ, USA). The culture media were incubated at 37 °C for 48 h, resulting in the growth of dry, creamy white colonies. The strain was determined as *Trichosporon inkin* by using the VITEK2 system (BioMérieux, Marcy l'Etoile, France). The strain was sent to Laboratório Especial de Micologia, UNIFESP, Brazil, for further analysis. Phylogenetic analysis of the intergenic spacer region (IGS1) of the ribosomal DNA placed the strain in a clade most closely related to *T. inkin*. The patient was successfully treated with fluconazole (400 mg/day) and was discharged from hospital 14 days after start of antifungal therapy.

### Additional *Trichosporon* Strains

An additional set of strains was included in our analysis, which was composed of: (a) six clinical *T. inkin* isolates stored in our Yeast Collection at LEMI-UNIFESP. The strains were collected from six patients admitted to different Brazilian medical centers between 2013 and 2019. They were selected for analysis because they fell within the same IGS1-clade as strain L9630 (= CBS 17435, ex-type); and (b) the type-strain of *T. inkin* CBS 5585 obtained from the CBS culture collection (Westerdijk Fungal Biodiversity Institute, Utrecht, The Netherlands); finally (c) after finishing the initial molecular analyses we became aware of similar strains from France, hence we included a set of 27 clinical strains recovered between 2006 and 2023 from 18 French hospitals. These French strains were initially identified as *Trichosporon* sp., closely related to *T. inkin,* at the French National Reference Center for invasive Mycoses and Antifungals, Institut Pasteur. Next to their IGS1-sequence data we included the antifungal susceptibility testing (see below) results for the azoles.

### DNA-Based Characterization and Phylogenetic Analysis

The strains were cultured onto malt extract agar (MEA; Oxoid, Basingstoke, United Kingdom) and incubated at 25 °C for 48 h. DNA extraction was carried out using the cetyltrimethylammonium bromide (CTAB) method as previously described [27]. The ribosomal DNA loci IGS1, internal transcribed spacer (ITS) and D1/D2 region of the LSU were amplified using the primers pair 26SF (5′-ATCCTTTGCAGACGACTTGA-3′) plus 5SR (5′-AGCTTGACTTCGCAGATCGG-3′) and V9G (5′-TTACGTCCCTGCCCTTTGTA-3′) plus LS266 (5′-GCATTCCCAAACAACTCGACTC-3′), respectively [11, 28]. DNA sequencing was conducted using the same set of primers in combination with the BigDye v3.1 sequencing kit and the ABI3130xL Genetic Analyzer platform (both from Applied Biosystems, Foster City, CA, USA). Raw data was assembled and edited using Phred–Phrap–Consed, targeting a Phred score of > 30 [29, 30]. The results were compared with sequences deposited in the NCBI GenBank database (http://ncbi.nlm.nih.gov) using the BLASTn tool.

The rDNA sequences were aligned via the online MAFFT platform and manually adjusted in MEGA v7. Additionally, an IGS1 sequence dataset for *T. inkin* was included in the analysis by collecting them from the NCBI GenBank database. Prior running the phylogenetic analysis, the best nucleotide substitution model was searched with MEGA v7 using the foreseen dataset as input, resulting in the Kimura-2-parameter with gamma distributed with invariant sites. These settings were used as options for the subsequent 1000× bootstrapped Maximum Likelihood (ML heuristic method = nearest-neighbor-interchange; gaps were treated as complete deletion) analysis in MEGA v7 [31–34].

Amplified fragment length polymorphism (AFLP) fingerprint analysis was performed as previously described (Lu et al., 2013), but by using the primer combinations HpyCH4IV (5′-FLU-GTAGACTGCGTACCCGTC-3′) and MseI (5′-GATGAGTCCTGACTAATGAT-3′). Fragment analysis was carried out on the ABI3730xL Genetic Analyzer platform (Applied Biosystems). Raw data was analyzed using BioNumerics v7.6 (Applied Math, St. Martems-Latum, Belgium), and a dendrogram was created using the Pearson correlation similarity coefficient and unweighted pair group method with arithmetic mean (UPGMA) cluster analysis algorithms [35].

### In Vitro Antifungal Susceptibility Testing

Antifungal susceptibility testing for fluconazole (FLC), voriconazole (VOR), isavuconazole (ISA), posaconazole (POS), and amphotericin B (AMB) (Sigma-Aldrich, St. Louis, MO, USA) was carried out following the EUCAST E.DEF 7.3.2 broth microdilution method [36]. The antifungal drugs were tested in concentration ranges of 0.125–64 mg/L for FLC and 0.03–16 mg/L for VRC, ISA, POS, and AMB. Minimal inhibitory concentrations (MIC) were determined after 48 h incubation, checking for the lower concentrations able to inhibit 50% of cell growth for azoles and 100% of growth inhibition for AMB [3, 36]. The reference strains *Pichia kudriavzevii* (syn. *Candida krusei*) ATCC 6258 (= CBS 573) and *Candida parapsilosis* ATCC 22019 (= CBS 604) were used as quality controls.

### Morphological and Physiological Characterization

Strain L9630 (= CBS 17435, ex-type) was further morphological and physiological characterized after growing it onto MEA, glucose yeast peptone agar (GYPA, Oxoid), and yeast morphology agar (YMoA, Difco). Plates were incubated at 25 °C for 7 days. Microscopic observations and measurements were carried out using an Axioskop 2 plus microscope (Carl Zeiss, Jena, Germany) fitted with a Nikon DS-Ri2 microscope camera (Nikon Instruments, Tokyo, Japan).

Physiological profiling was conducted using standard methods commonly employed in yeast taxonomy [37]. Nitrogen and carbon compound utilization was assessed by auxanograms and liquid media, respectively. The growth profile at different temperatures was evaluated using GYPA plates for up to 1 week.

In addition, we assessed the identification of the strain by performing matrix-assisted laser desorption–ionization-time of flight mass spectrometry (MALDI–TOF MS) using the Biotyper database v.4.1 (Bruker Daltonics, Bremen, Germany). Main spectra (MSP) were created as previously published [38, 39].

## Results

### DNA-Based Characterization and Species Delineation

Based on IGS1-sequencing, strain L9630 (= CBS 17435, ex-type) and the six related *Trichosporon* isolates that were selected for the genetic analysis displayed a low identification score when compared by BLAST with data in the NCBI Nucleotide database, showing a low similarity score of 81% with *T. inkin*. The D1/D2 LSU sequences had > 99% similarity with that of *T. inkin*, *T. dohaense*, *T. lactis*, and *T. ovoides*.

**Fig. 1:**
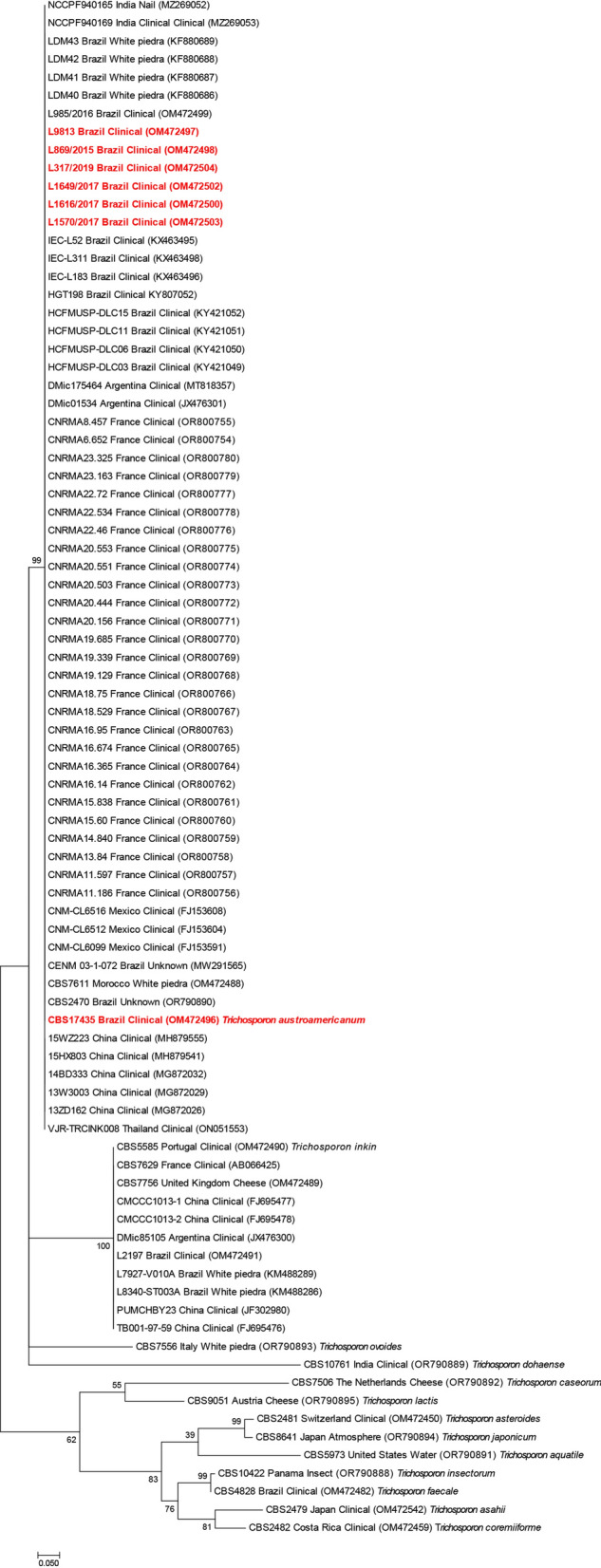
Phylogenetic tree of the here described novel species *Trichosporon austroamericanum* and currently recognized species within the *Trichosporon* genus based on IGS1-sequences. The phylogenetic tree was drawn based on a 1000× bootstrap Maximum Likelihood analysis. The tree with the highest log likelihood (− 914.03) is shown. The percentage of trees in which the associated taxa clustered together is shown next to the branches. The tree is drawn to scale, with branch lengths measured in the number of nucleotide substitutions per site. All positions containing gaps and missing data were eliminated. There were a total of 113 positions in the final dataset. Data in bold indicates strains genotypically and phenotypically characterized in the current study. Strain numbers are followed by information about the country of origin, source and NCBI Genbank accession number

Phylogenetic analysis using IGS1-sequences data from our seven clinical strains along with data from Institut Pasteur and the NCBI GenBank database encompassing all currently recognized species within the genus *Trichosporon*. In addition, we incorporated into our analyses all available IGS1-sequences of* T*. *inkin* deposited by several diagnostic reference laboratories. The IGS1 region analysis was chosen due to its superior capability in distinguishing phylogenetically related species when compared to the ITS sequence, as previously described [11]. Our findings consistently demonstrated that DNA sequences of strain L9630 (= CBS 17435, ex-type) and its six Brazilian and 27 French siblings had identical IGS1-sequences and yield a clearly defined and well-supported clade. This further supports our observation of a novel species, hereafter designated as *Trichosporon austroamericanum* (Fig. 1). As depicted by the 1,000× bootstrap Maximum Likelihood analysis, *T. austroamericanum* (L9630 = CBS 17435, ex-type) clustered most closely with *T. inkin* (CBS 5585^ T^) followed distantly with *T. ovoides* and *T. dohaense* (Fig. 1). The recognition of *T. austroamericanum* as a distinct lineage within the genus *Trichosporon* was further substantiated through analysis of the commonly used ITS region (Table 1). Maximum likelihood phylogeny showed strong bootstrap support of 81%, respectively, although the species was found to be closely related to *T. inkin* (Supplementary Information 1) (Table 1).

**Table 1:**
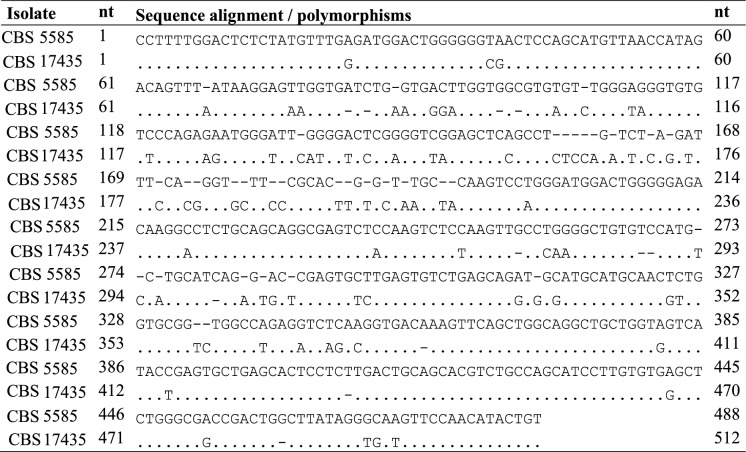
Polymorphic sites of the intergenic spacer 1 (IGS1) ribosomal DNA of *Trichosporon austroamericanum* CBS 17435 (ex-type) compared to *T. inkin* CBS 5585^ T^

**Fig. 2:**
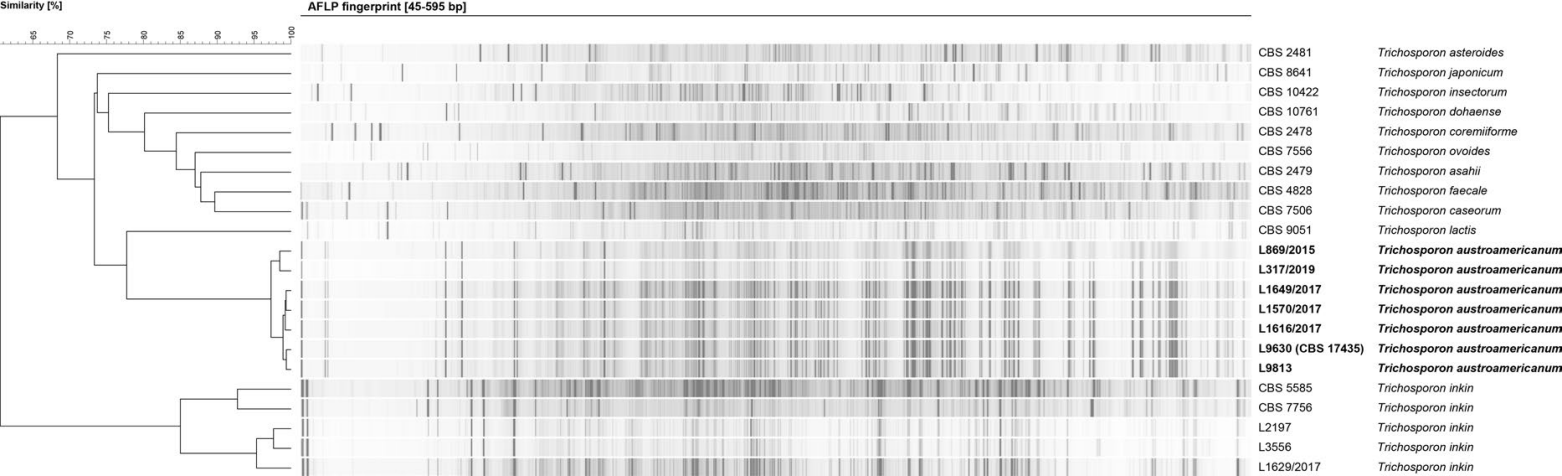
AFLP-fingerprint analysis of the *Trichosporon* genus. The strain L9630 (= CBS 17435, ex-type) and their six related clinical strains (L9813, L869/2015, L1649/2017, L1570/2017, L1616/2017, and L317/2019) exhibited an 97% fingerprint similarity, exhibiting only 61% similarity compared with the fingerprints of *T. inkin* type strain, 73% similarity with *T. asteroides*, and 78% similarity with *T. lactis*

By AFLP-fingerprinting analysis, the L9630 (= CBS 17435, ex-type) strain and their six related clinical strains (Table 2) shared an 97% fingerprint similarity, exhibiting only 61% similarity compared with the fingerprints of *T. inkin* type strain, 73% similarity with *T. asteroides*, and 78% similarity with *T. lactis* (Fig. 2).

**Table 2:** In vitro antifungal susceptibility of seven clinical *Trichosporon austroamericanum* strains

Strain	Sources	Antifungals MICs (mg/L)				
		Fluconazole	Voriconazole	Isavuconazole	Posaconazole	Amphotericin B
CBS 17435	Bladder puncture	4	0.03	0.03	0.06	4
L9813	Lung biopsy	4	0.03	0.125	0.25	2
L869/2015	Urine	4	0.03	0.03	0.06	4
L1649/2017	Blood culture	2	0.06	0.06	0.25	8
L1570/2017	Surgical wound	4	0.03	0.03	0.03	1
L1616/2017	Piedra branca	4	0.03	0.03	0.25	2
L317/2019	Urine	2	0.03	0.03	0.06	2

Antifungal susceptibility testing performed according to EUCAST E.DEF 7.3.2 broth microdilution method, with readings performed after 48 h of incubation [36]. CBS 17435, ex-type strain; MIC, minimal inhibitory concentration

### In Vitro Antifungal Susceptibility Testing

Table 2 summarizes the MIC values (mg/L) obtained from the seven Brazilian clinical *Trichosporon* strain tested. The highest MIC values were obtained for amphotericin B (1–8 mg/L), followed by fluconazole (2–4 μg/ml), posaconazole (0.03–0.25 mg/L), isavuconazole (0.03–0.125 mg/L), and voriconazole (0.03–0.06 mg/L).

Regarding the 27 strains obtained from the French collection and tested against four azole compounds, the highest MIC values were observed for fluconazole (0.25–4 mg/L). Following closely were isavuconazole (0.03–4 mg/L), posaconazole (0.03–0.25 mg/L), and voriconazole (0.03–0.125 mg/L; Supplementary Information 2).

### Morphological and Physiological Characterization

**Fig. 3:**
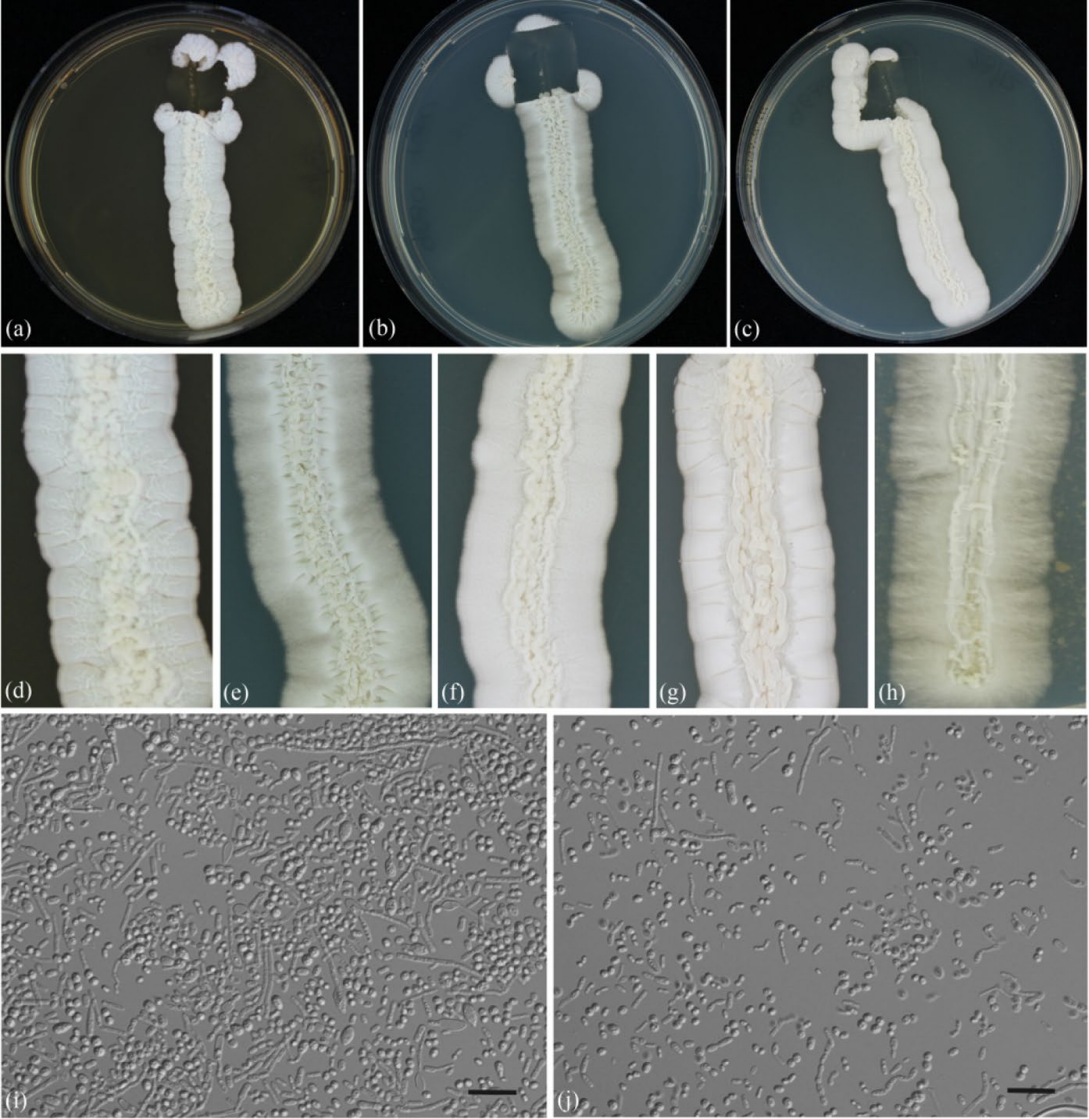
*Trichosporon austroamericanum* ex-type CBS 17435^ T^. Colonies on, from left to right, MEA (**a**), YMoA (**b**), and GYPA (**c**). Colony close‐ups on: MEA (**d**), YMoA (**e**), GYPA (**f**), YMA (**g**) and PDA (**h**). **i**, **j** Yeast morphology on MEA with blastoconidia, hypha and arthroconidia observed by light microscopy. Scale bars: (i–j) = 10 µm

The six isolates displayed the typical morphology of *Trichosporon* (Fig. 3). On MEA plates, colonies exhibited a cream to beige color, with a membranous, friable, and farinose texture. Colonies were raised, rough, featuring transversal/cerebriform marginal zones, along with a fringed appearance (Fig. 3a, d). Likewise, on YMoA plates, the same mentioned characteristics were present, besides colonies showing a flat lateral zone with transversal lines (Fig. 3e). Compared to *T. inkin*, *T. austroamericanum* exhibits distinctive characteristics, such as delayed but pronounced d-glucosamine and melibiose assimilation, weakly positive l-arabinose utilization, lack of growth on 50% glucose, resilience to 0.1% cycloheximide, and the ability to thrive at temperatures up to 45 °C (Table 3). Identification performed by MALDI–TOF MS did not yield any matching patterns (score values ranging from 0.00 to 1.69). The closest match generated by spectrometry was *T. inkin* with a score value of 1.68 for CBS 5585. Consequently, new MALDI-TOF MS main spectra (MSPs) were generated for the new species. The MSP was incorporated at the WI-KNAW in-house library, ensuring reliable identification through Bruker Daltonics MALDI–TOF MS, as well as to CDC-hosted MicrobeNet (https://www.cdc.gov/microbenet/).

**Table 3:** *Trichosporon austroamericanum* physiological characteristics

Species characteristics	*T. inkin*	*T. austroamericanum*						
	CBS 5585^ T^*	CBS 17435^ T^**	L9813	L869/2015	L317/2019	L1649/2017	L1570/2017	L1616/2017
*Fermentation*								
d-Glucose	−	−	−	−	−	−	−	−
*Growth reactions and other characteristics*								
d-Glucose	+	+	+	+	+	+	+	+
d-Galactose	+	+	+	+	+	+	+	+
l-Sorbose	−	+	+	+	+	+	s, +	+
d-Glucosamine	−	d, +	+	+	+	+	w	+
d-Ribose	+	+	+	+	+	+	+	+
d-Xylose	+	+	+	+	+	+	+	+
l-Arabinose	−	w, s	d, +	+	w	w	w, s	w
d-Arabinose	−	+	+	+	+	+	+	+
l-Rhamnose	−	d, +	d, +	+	w	w	w	d, +
Sucrose	+	+	+	+	+	+	+	+
Maltose	+	+	+	+	+	+	+	+
α,α-Trehalose	+	+	+	+	+	+	+	+
Methyl α-d-glucoside	+	+	+	+	+	+	+	+
Cellobiose	+	+	+	+	+	+	+	+
Salicin	d	s, +	s, +	+	d, +	+	d, +	d, +
Arbutin	+	+	+	+	+	+	+	+
Melibiose	−	d, +	d, +	w	w	d, +	d, +	w
Lactose	+	+	+	+	+	+	+	+
Raffinose	−	w	w	w	w	w	w	w
Melezitose	+	+	+	s, +	+	+	+	+

	CBS 5585^ T^	CBS 17435^T^	L9813	L869/2015	L317/2019	L1649/2017	L1570/2017	L1616/2017
Inuline	−	w, s	w	w	s, +	w	w	w
Soluble starch	+	+	+	+	+	+	+	+
Glycerol	−, d, w	+	+	+	d, +	+	+	+
*meso*-Erythritol	+	+	+	+	+	+	+	+
Ribitol	−	w, s	d, +	w, s	w	w	w	w, s
Xylitol	−	w, s	d, +	+	w	w	w	d, +
l-arabinitol	−	w, s	+	+	w	w	w, s	+
d-glucitol	−	w, s	w	w, s	w	w	w	w
d-mannitol	+	+	+	+	+	+	w, s	+
Galactitol	−	w	w	w	w	w	w	w
*myo*-Inositol	+	+	+	+	+	+	+	+
Glucono-δ-lactone	−	−	−	−	−	−	−	−
2-Keto-d-Gluconate	+	+	+	+	+	+	+	+
d-Gluconate	+	+	+	+	+	+	+	+
d-Glucuronate	+ , d, w	+	+	+	+	+	+	+
d-Galacturonate	−	−	−	−	−	−	−	−
dl-Lactate	+	+	+	+	+	+	+	+
Succinate	d	−	−	−	−	−	−	−
Citrate	d	−	−	−	−	−	−	−
Methanol	−	w	w	w	w	w	w	w
Ethanol	+	+	+	+	+	+	+	+
Propane 1,2 diol	+	+	+	+	+	+	+	+
Butane 2,3 diol	−, d, w	w	w	+	w	w	w	w
Quinic acid	−	−	−	−	−	−	−	−
Saccharate	nd	w	w	w	w	w	w	w
Galactonic acid	+	+	+	+	+	+	+	+
Nitrate	−	−	−	−	−	−	−	−
Nitrite	** + **	−	−	−	−	−	−	−
Ethylamine	+	+	+	+	+	+	+	+
l-lysine	+	+	+	+	+	+	+	+
Cadaverine	+	+	+	+	+	+	+	+
Creatine	−	−	−	−	−	-	−	−
Creatinine	−	−	−	−	−	−	−	−
Glucosamine	−	−	−	−	−	−	−	−
Imidazole	−	−	−	−	−	−	−	−
d-Tryptophan	−	−	−	−, w	−	−	−, w	−
Proline	nd	−, w	−	−, w	−	−	−	−
Osmotolerance (50% glucose)	** + **	−	−	−	−	−	−	−
Starch production	−	−	−	−	−	−	−	−
Urea test	+	+	+	+	+	+	+	+
DBB reaction	+	+	+	+	+	+	+	+
Tolerance to cycloheximide 0.01% (w/v)	−, d, w	+	+	+	+	+	+	+
Tolerance to cycloheximide 0.1% (w/v)	−	+	+	+	+	+	+	+
Growth at 4 °C	nd	−	−	−	−	−	−	−
Growth at 10 °C	nd	( +)	( +)	−	−	−	−	−
Growth at 18 °C	nd	+	+	+	+	+	+	+
Growth at 21 °C	nd	+	+	+	+	+	+	+
Growth at 25 °C	+	+	+	+	+	+	+	+
Growth at 30 °C	+	+	+	+	+	+	+	+
Growth at 37 °C	+	+	+	+	+	+	+	+
Growth at 42 °C	+	+	+	+	+	+	+	+
Growth at 45 °C	−	+	+	+	+	+	+	+

^*^Physiological data for reference strain CBS 5575^ T^ were obtained from the CBS Culture Collection Database (https://wi.knaw.nl/page/Collection). ^**^CBS 17435 ex−type strain; Glucose was used to ensure the inability of *Trichosporon* species to ferment compounds. *Trichosporon inkin* displayed a restrictive physiology when compared to *T. austroamericanum* strains. Both species grew at a temperature ranging from 25 to 42 °C, and the *T. austroamericanum* was able to grow at 45 °C. Data were scored according to [37, 36]: + , positive; − , negative; d, late; s, slow positive; w, weakly positive; ( +), seldom positive; nd, no data available; DBB reaction: diazonium blue b staining reaction

The main growth requirements able to distinguish strain L9630 (= CBS 17435, ex-type) and their six relatives strains tested from *T. inkin* can be summarized as follows: capability to grow at 45 °C; utilization of l-sorbose, d-glucosamine, l-arabinose, d-arabinose, melibiose, inulin, methanol, and other sources detailed in Table 3, although growth may be weak or delayed for some; inability to use both succinate and citrate; and the species does not grow in media with elevated sugar concentration (50% glucose). Considering that physiological characterization can be susceptible to subjectivity in result interpretation, sequencing of the IGS1 region or application of MALDI–TOF MS provide a dependable tool for the precise identification of *T. austroamericanum*.

Here an integrated analysis, which incorporated a combination of morphology, physiology, and phylogeny, was employed to define our new species and formally describe it as *T. austroamericanum* below.

## Discussion

The phylogenetic analysis and AFLP fingerprinting, that included the strain L9630 (= CBS 17435, ex-type) and an additional set of clinical *Trichosporon* strains obtained from our laboratory, showed that all these strains were closely related to *T. inkin,* but grouped together in a different clade. In addition, these strains displayed distinctive physiological characteristics when compared to *T. inkin,*

The integration of morphological, physiological, and genetic analyses based on rDNA targets (ITS, IGS1) and AFLP-fingerprint provided a comprehensive characterization of the new species. The new clinically relevant yeast species *Trichosporon austroamericanum* (= CBS 17435, ex-type culture) was isolated from a urine sample collected from a kidney transplant recipient hospitalized at a Brazilian tertiary care center. The morphological observations on different growth media cosubstantiate the typical characteristics of *Trichosporon* species, without any morphological finding able to distinguish between the *Trichosporon* species. On the other hand, physiological profiling of strain L9630 (= CBS 17435, ex-type) could distinguish it from *T. inkin* as further detailed in Table 3 [9]. Given the intrinsic resistances of basidiomycetous yeasts to echinocandins, this holds true for *T. austroamericanum* too.

The IGS1 region has been used in the characterization of *Trichosporon* species, emphasizing its significance in distinguishing potentially identical species that otherwise might appear genetically identical when using other rDNA regions, like ITS. The characterization of *Trichosporon mycotoxinivorans* was performed by phylogenetic analysis using different rDNA regions, the species exhibited high homology with *T. loubieri* for the ITS and D1/D2 loci. However, analysis of the commonly used IGS marker successfully differentiated both species, which were otherwise difficult to discriminate from each other using ITS and D1/D2 data. By IGS analysis these two species had only 66.7% similarity [40]. Similarly, in the characterization of *T. terricola*, although D1/D2, ITS, and IGS sequences effectively placed the new species into a distinct clade, the nucleotide differences observed in the IGS1 region played a pivotal role in conclusively identifying the studied isolate as a species [41]. We observed that the D1/D2 sequence of *T. austroamericanum* was > 99% similar to *T. inkin*, *T. dohaense*, *T. lactis*, and *T. ovoides*, showing that this part of the ribosomal DNA is not suitable to identify *Trichosporon* species, while IGS has a superior ability to do so [11].

Considering the occurrence of the novel species, BLAST searches and a subsequent phylogenetic analysis, showed that *T. austroamericanum* has been isolated from diverse clinical samples (Fig. 1), including clones obtained from invasive infections (MG872032, China), urine (ON051553, Thailand), blood (KX463498, Brazil), superficial mycosis (MZ269052, India), and others human sources (JX476301, Argentina; Fig. 1). Retrospectively, we identified the first clinical strains recovered in France in 2006 from an African immigrant. These findings underscore the apparent global distribution of this pathogen and the urgent requirement for further studies to characterize the burden, natural history, and potential therapeutic peculiarities of *T. austroamericanum* infections.

In conclusion, the identification and characterization of a novel species named *T. austroamericanum* contribute to the knowledge of the pathogenic species belonging to the basidiomycetous yeast genus *Trichosporon*. Nevertheless, additional investigations are warranted to fully uncover the extent of *T. austroamericanum* infections and their implications for public health.

## Taxonomy

### *Trichosporon austroamericanum,* E.C. Francisco, A.L. Colombo, M. Desnos-Ollivier & F. Hagen, sp. nov.

MycoBank MB 850273 (Fig. 3).

### Etymology

Latin, *austro.americanum*, referring to the South American continent, the geographical area where the new species was first described.

### Typus

Holotype CBS H-24937, ex‐type culture CBS 17435 = L9630 = 2MG-A1202-62. Brazil, São Paulo, from a urine sample, 2013, and stored at Yeast Collection at the LEMI-UNIFESP and investigated by Elaine C. Francisco.

### Barcode Sequences

Intergenic spacer (IGS1) ribosomal DNA locus = OM472496.

Internal Transcibed Spacer (ITS) ribosomal DNA locus = PP192021.

D1/D2 region of the large subunit (LSU) ribosomal DNA locus = PP508390.

### Description

**Growth on GYPA:** after 7 days at 25 °C, colonies are cream to beige colored, membranous, friable, rough, raised, with a featuring transversal/cerebriform marginal zones, and a slightly fissured margin, measuring 3628.798 μm (Fig. 3c, f). **Growth on MEA:** after 7 days at 25 °C, colonies exhibit similar features as on GYPA, but have a farinose texture (Fig. 3a, d). Septate hyphae abundant (cells length × width 15 – 33 × 1 – 2.5 μm), with several globose to ellipsoidal blastoconidia (2 – 4 × 1.5 – 3 μm in diameter) present; long cylindrical arthroconidia present. Growth on GYPA and MEA: appressoria are absent in slide cultures. Growth on yeast morphology agar: after 1 week at 25 °C, colony ca. 9–13 mm wide, exhibiting a similar morphology on GYPA and MEA media, including a strong irregular and ridged lateral zone (Fig. 3b, e).

#### Nutritional Data and Growth Temperatures

*Trichosporon austroamericanum* exhibits delayed but pronounced d-glucosamine and melibiose assimilation, weakly positive l-arabinose utilization, lack of growth on 50% glucose, resilience to 0.1% cycloheximide, and the ability to thrive at temperatures up to 45 °C. Urease-positive. The full dataset of physiological characteristics is provided in Table 3.

#### Ecology, and Occurrence

*Trichosporon austroamericanum* is often isolated from clinical specimens, but its environmental ecological niche is unknown.

The holotype CBS H-24937 was isolated from a urine sample obtained via an ultrasound-guided percutaneous puncture performed in a Brazilian kidney transplant recipient with an abdominal collection secondary to urinary fistula. Based on intergenic spacer (IGS1) ribosomal DNA locus available via the NCBI GenBank database, *T. austroamericanum* has been isolated from China, France, India, and Argentina, so far only from clinical sources.

#### Identification and Phenotypical Differences with Closely Related Species

*Trichosporon austroamericanum* can be distinguished from the other species belonging to the *Trichosporon* genus by sequencing the intergenic spacer (IGS1) ribosomal DNA locus. The expansion of MALDI–TOF MS databases has enabled the accurate identification of this species and has become a powerful tool capable of distinguishing *T. austroamericanum* from *T. inkin*. Furthermore, *T. austroamericanum* displays a broader range of physiological characteristics compared to *T. inkin*, including the ability to thrive at 45 °C.

### Supplementary Information

Below is the link to the electronic supplementary material.

Supplementary file1 (DOCX 22 KB)

Supplementary file2 (PDF 191 KB)

Supplementary file3 (JPG 1006 KB)

## Data Availability

The strain L9630 (= CBS 17435, ex-type; CBS H-24937, holotype) has been deposited in the CBS culture collection, hosted at the Westerdijk Fungal Biodiversity Institute, Utrecht, The Netherlands. The generated sequences have been deposited in the NCBI GenBank repository. The strains and sequence data accession numbers are indicated throughout the manuscript, all accession numbers are provided in Fig. 2. Additionally, the MALDI TOF MS main spectra (MSP) of CBS 17435 (ex-type) have been integrated into the in-house database of the Westerdijk Fungal Biodiversity Institute as well as to CDC’s MicrobeNet (https://www.cdc.gov/microbenet/). NCBI GenBank accession numbers for the here generated IGS sequence data: OM472496–OM472498, OM472500, and OM472502–OM472504, OR790888-OR790895, and OR800754–OR800780; for ITS sequence data: PP192020 and PP192021; for D1/D2 sequence data: PP508390-PP508395. Additionally, for all IGS and ITS data used from previously submitted data to NCBI Genbank, the accession numbers are also provided in Fig. 1 and the Supplementary Figure 1.

## References

[1] Arastehfar A, de Almeida Júnior JN, Perlin DS, Ilkit M, Boekhout T, Colombo AL (2021). Multidrug-resistant *Trichosporon* species: underestimated fungal pathogens posing imminent threats in clinical settings. Crit Rev Microbiol.

[2] Francisco EC, de Almeida Junior JN, Queiroz-Telles F, Aquino VR, Mendes AVA, de Oliveira SM, Castro PTOE, Guimarães T, Ponzio V, Hahn RC, Chaves GM, Colombo AL (2021). Correlation of *Trichosporon asahii* genotypes with anatomical sites and antifungal susceptibility profiles: data analyses from 284 isolates collected in the last 22 years across 24 medical centers. Antimicrob Agents Chemother.

[3] Francisco EC, de Almeida Junior JN, de Queiroz TF, Aquino VR, Mendes AVA, de Andrade Barberino MGM, de Tarso O, Castro P, Guimarães T, Hahn RC, Padovan ACB, Chaves GM, Colombo AL (2019). Species distribution and antifungal susceptibility of 358 *Trichosporon* clinical isolates collected in 24 medical centres. Clin Microbiol Infect.

[4] Colombo AL, Padovan ACB, Chaves GM (2011). Current knowledge of *Trichosporon* spp. and trichosporonosis. Clin Microbiol Rev.

[5] Turunen J, Paalanne N, Reunanen J, Tapiainen T, Tejesvi MV (2023). Development of gut mycobiome in infants and young children: a prospective cohort study. Pediatr Res.

[6] Nobrega de Almeida J, Francisco EC, Holguín Ruiz A, Cuéllar LE, Rodrigues Aquino V, Verena Mendes A, Queiroz-Telles F, Santos DW, Guimarães T, Maranhão Chaves G, Grassi de Miranda B, Araújo Motta F, Vargas Schwarzbold A, Oliveira M, Riera F, Sardi Perozin J, Pereira Neves R, França E Silva ILA, Sztajnbok J, Fernandes Ramos J, Borges Botura M, Carlesse F, de Tarso de O E Castro P, Nyirenda T, Colombo AL (2021). Epidemiology, clinical aspects, outcomes and prognostic factors associated with *Trichosporon* fungaemia: results of an international multicentre study carried out at 23 medical centres. J Antimicrob Chemother.

[7] Chin VK, Yong VC, Chong PP, Amin Nordin S, Basir R, Abdullah M (2020). Mycobiome in the gut: a multiperspective review. Mediators Inflamm.

[8] Guo LN, Yu SY, Hsueh PR, Al-Hatmi AMS, Meis JF, Hagen F, Xiao M, Wang H, Barresi C, Zhou ML, de Hoog GS, Xu YC (2019). Invasive infections due to *Trichosporon*: species distribution, genotyping, and antifungal susceptibilities from a multicenter study in China. J Clin Microbiol.

[9] Sugita T, Kurtzman C, Fell JW, Boekhout T (2011). *Trichosporon* Behrend (1890). The Yeasts, A Taxonomic study.

[10] Guého E, Smith MT, de Hoog GS, Billon-Grand G, Christen R, Batenburg-van der Vegte WH (1992). Contributions to a revision of the genus *Trichosporon*. Antonie Van Leeuwenhoek.

[11] Sugita T, Nakajima M, Ikeda R, Matsushima T, Shinoda T (2002). Sequence analysis of the ribosomal DNA intergenic spacer 1 regions of *Trichosporon* species. J Clin Microbiol.

[12] Sugita T, Takashima M, Nakase T, Ichikawa T, Shinoda T, Nishikawa A (2002). A basidiomycetous anamorphic yeast, *Trichosporon terricola* sp. nov., isolated from soil. J Gen Appl Microbiol.

[13] Motaung TE, Albertyn J, Kock JL, Lee CF, Suh SO, Blackwell M, Pohl CH (2013). *Trichosporon vanderwaltii* sp. nov., an asexual basidiomycetous yeast isolated from soil and beetles. Antonie Van Leeuwenhoek.

[14] Liu XZ, Wang QM, Göker M, Groenewald M, Kachalkin AV, Lumbsch HT, Millanes AM, Wedin M, Yurkov AM, Boekhout T, Bai FY (2015). Towards an integrated phylogenetic classification of the *Tremellomycetes*. Stud Mycol.

[15] Liu XZ, Theelen B, Groenewald M, Bai FY, Boekhout T (2015). Phylogeny of tremellomycetous yeasts and related dimorphic and filamentous basidiomycetes reconstructed from multiple gene sequence analyses. Stud Mycol.

[16] Takashima M, Manabe RI, Nishimura Y, Endoh R, Ohkuma M, Sriswasdi S, Sugita T, Iwasaki W (2019). Recognition and delineation of yeast genera based on genomic data: Lessons from *Trichosporonales*. Fungal Genet Biol.

[17] Takashima M, Sugita T (2019). Draft genome analysis of *Trichosporonales* species that contribute to the taxonomy of the genus *Trichosporon* and related taxa. Med Mycol J.

[18] Takashima M, Kurakado S, Cho O, Kikuchi K, Sugiyama J, Sugita T (2020). Description of four *Apiotrichum* and two *Cutaneotrichosporon* species isolated from guano samples from bat-inhabited caves in Japan. Int J Syst Evol Microbiol.

[19] Kuo SH, Lu PL, Chen YC, Ho MW, Lee CH, Chou CH, Lin SY (2021). The epidemiology, genotypes, antifungal susceptibility of *Trichosporon* species, and the impact of voriconazole on *Trichosporon* fungemia patients. J Formos Med Assoc.

[20] Dabas Y, Xess I, Kale P (2017). Molecular and antifungal susceptibility study on trichosporonemia and emergence of *Trichosporon mycotoxinivorans* as a bloodstream pathogen. Med Mycol.

[21] Santos FA, Leite-Andrade MC, Vasconcelos MA, Alves AI, Buonafina-Paz MD, Araújo-Neto LN, Macêdo DP, Neves RP (2022). *Trichosporon inkin* fungemia case report: clinical and laboratory management. Future Microbiol.

[22] Rodriguez-Tudela JL, Diaz-Guerra TM, Mellado E, Cano V, Tapia C, Perkins A, Gomez-Lopez A, Rodero L, Cuenca-Estrella M (2005). Susceptibility patterns and molecular identification of *Trichosporon* species. Antimicrob Agents Chemother.

[23] Arabatzis M, Abel P, Kanellopoulou M, Adamou D, Alexandrou-Athanasoulis H, Stathi A, Platsouka E, Milioni A, Pangalis A, Velegraki A (2014). Sequence-based identification, genotyping and EUCAST antifungal susceptibilities of *Trichosporon* clinical isolates from Greece. Clin Microbiol Infect.

[24] Liu Q, Wang X (2021). Characterization and phylogenetic analysis of the complete mitochondrial genome of pathogen *Trichosporon inkin* (*Trichosporonales*: *Trichosporonaceae*). Mitochondrial DNA B Resour.

[25] de Aguiar CR, Serpa R, Flávia Uchoa Alexandre C, de Farias Marques FJ, Silva V, de Melo C, da Silva FJ, de Jesus J, Evangelista A, Pires de Camargo Z, Samia Nogueira Brilhante R, Fabio Gadelha Rocha M, Luciano Bezerra Moreira J, de Jesus Pinheiro Gomes Bandeira T, Júlio Costa Sidrim J (2015). *Trichosporon inkin* biofilms produce extracellular proteases and exhibit resistance to antifungals. J Med Microbiol.

[26] Iturrieta-González IA, Padovan AC, Bizerra FC, Hahn RC, Colombo AL (2014). Multiple species of *Trichosporon* produce biofilms highly resistant to triazoles and amphotericin B. PLoS ONE.

[27] Hoog GS, Ende AHGG (1998). Molecular diagnostics of clinical strains of filamentous Basidiomycetes. Mycoses.

[28] Gerrits van den Ende AHG, de Hoog GS (1999). Variability and molecular diagnostics of the neurotropic species *Cladophialophora bantiana*. Stud Mycol.

[29] Ewing B, Green P (1998). Base-calling of automated sequencer traces using phred II error probabilities. Genome Res.

[30] Gordon D (2003). Viewing and editing assembled sequences using Consed. Curr Protoc Bioinform.

[31] Katoh K, Rozewicki J, Yamada KD (2019). MAFFT online service: multiple sequence alignment, interactive sequence choice and visualization. Brief Bioinform.

[32] Edgar RC (2004). MUSCLE: multiple sequence alignment with high accuracy and high throughput. Nucleic Acids Res.

[33] Kimura M (1980). A simple method for estimating evolutionary rates of base substitutions through comparative studies of nucleotide sequences. J Mol Evol.

[34] Saitou N, Nei M (1987). The neighbor-joining method: a new method for reconstructing phylogenetic trees. Mol Biol Evol.

[35] Lu XL, Najafzadeh MJ, Dolatabadi S, Ran YP, Gerrits van den Ende AH, Shen YN, Li CY, Xi LY, Hao F, Zhang QQ, Li RY, Hu ZM, Lu GX, Wang JJ, Drogari-Apiranthitou M, Klaassen C, Meis JF, Hagen F, Liu WD, de Hoog GS (2013). Taxonomy and epidemiology of *Mucor irregularis*, agent of chronic cutaneous mucormycosis. Persoonia.

[36] Arendrup MC, Meletiadis J, Mouton JW, Lagrou K. EUCAST Definitive Document E.DEF 7.3.2 Method for the determination of broth dilution minimum inhibitory concentrations of antifungal agents for yeasts. 2020. 10.1111/j.1469-0691.2012.03880.x 22563750

[37] Kurtzman CP, Fell JW, Boekhout T, Robert V. Methods for isolation, phenotypic characterization and maintenance of yeasts. In: The Yeasts. Elsevier, 87–110. 10.1016/B978-0-444-52149-1.00007-0

[38] Francisco EC, Ebbing M, Colombo AL, Hagen F, Trichosporon Brazilian Network (2023). Identification of clinical * Trichosporon asteroides * strains by MALDI-TOF mass spectrometry: evaluation of the Bruker Daltonics commercial system and an in-house developed library. Mycopathologia.

[39] Kolecka A, Khayhan K, Groenewald M, Theelen B, Arabatzis M, Velegraki A, Kostrzewa M, Mares M, Taj-Aldeen SJ, Boekhout T (2013). Identification of medically relevant species of arthroconidial yeasts by use of matrix-assisted laser desorption ionization-time of flight mass spectrometry. J Clin Microbiol.

[40] Molnar O, Schatzmayr G, Fuchs E, Prillinger H (2004). *Trichosporon mycotoxinivorans* sp. nov., a new yeast species useful in biological detoxification of various mycotoxins. Syst Appl Microbiol.

[41] Sugita T, Takashima M, Nakase T, Ichikawa T, Shinoda T, Nishikawa A (2002). A basidiomycetous anamorphic yeast, *Trichosporon terricola* sp. nov., isolated from soil. J Gen Appl Microbiol.

