# Supplementary material for: Unveiling Trichosporon austroamericanum sp. nov.: A Novel Emerging Opportunistic Basidiomycetous Yeast Species
Source: Mycopathologia. 2024 May 6;189(3):43. doi: 10.1007/s11046-024-00851-4 (PMC11074034; doi:10.1007/s11046-024-00851-4)
Supplement: Supplementary file 1 — Supplementary file1 (DOCX 22 KB) [file 11046_2024_851_MOESM1_ESM.docx]

**Supplementary information 1.** Phylogenetic tree of *Trichosporon* type strains and species from the related taxa *Apiotrichum* and *Cutaneotrichosporon* (major clinical lineages) obtained by maximum-likelihood analysis of ITS region with 1,000× bootstrap. The tree with the highest log likelihood (-1341.88) is shown. The percentage of trees in which the associated taxa clustered together is shown next to the branches. Initial tree(s) for the heuristic search were obtained automatically by applying Neighbor-Join and BioNJ algorithms to a matrix of pairwise distances estimated using the Maximum Composite Likelihood approach, and then selecting the topology with superior log likelihood value. The tree is drawn to scale, with branch lengths measured in the number of substitutions per site. This analysis involved 23 nucleotide sequences, All positions containing gaps and missing data were eliminated. There was a total of 428 positions in the final dataset. Note: the NCBI accession numbers are indicated between brackets after the CBS strain accession numbers.
