# Supplementary material for: Unveiling Trichosporon austroamericanum sp. nov.: A Novel Emerging Opportunistic Basidiomycetous Yeast Species
Source: Mycopathologia. 2024 May 6;189(3):43. doi: 10.1007/s11046-024-00851-4 (PMC11074034; doi:10.1007/s11046-024-00851-4)
Supplement: Supplementary file 2 — Supplementary file2 (PDF 191 KB) [file 11046_2024_851_MOESM2_ESM.pdf]

Supplementary information 2. *In vitro* antifungal susceptibility of 27 seven *Trichosporon austroamericanum* strains from the French collection

**Antifungals**	**MIC_{50} (mg/l)**	**MIC_{90} (mg/L)**	**Range (mg/L)**
Fluconazole	0.5	4	0.25-4
Voriconazole	0.03	0.06	0.015-0.125
Posaconazole	0.06	0.25	0.015-0.25
Isavuconazole	0.03	0.125	0.015-4

MIC, minimal inhibitory concentration; MIC_{50}, minimum inhibitory concentration required to inhibit the growth of 50% of organisms; MIC_{90}, minimum inhibitory concentration required to inhibit the growth of 90% of organisms.
