# Supplementary figures and images for: Unveiling Trichosporon austroamericanum sp. nov.: A Novel Emerging Opportunistic Basidiomycetous Yeast Species
Source: Mycopathologia. 2024 May 6;189(3):43. doi: 10.1007/s11046-024-00851-4 (PMC11074034; doi:10.1007/s11046-024-00851-4)

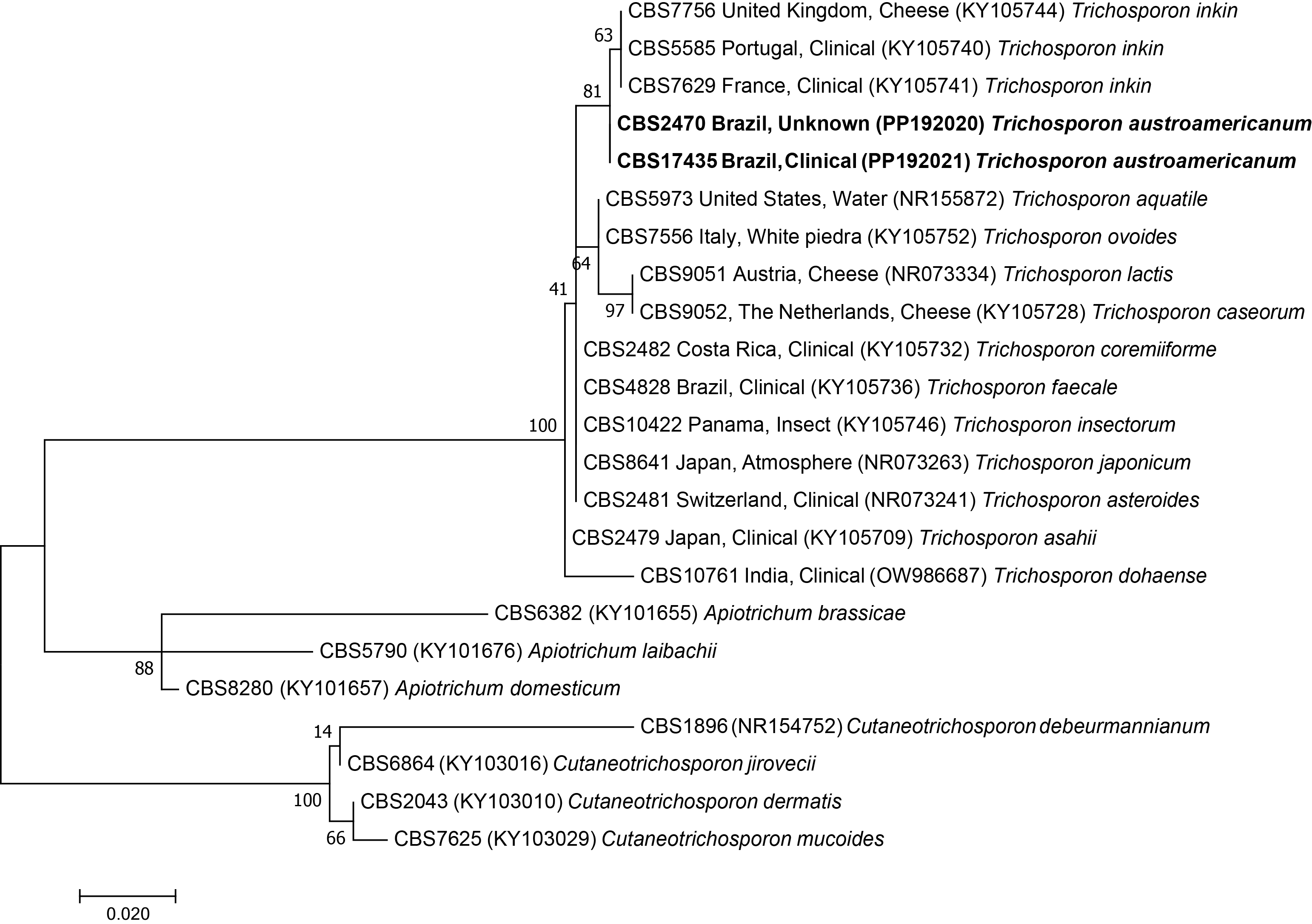

Supplement: Supplementary file 3 — Supplementary file3 (JPG 1006 KB) [file 11046_2024_851_MOESM3_ESM.jpg]
